# Skeletal muscle differentiation induces wide-ranging nucleosome repositioning in muscle gene promoters

**DOI:** 10.1038/s41598-024-60236-x

**Published:** 2024-04-24

**Authors:** Sonalí Harris, Iqra Anwar, Syeda S. Baksh, Richard E. Pratt, Victor J. Dzau, Conrad P. Hodgkinson

**Affiliations:** Mandel Center for Heart and Vascular Research, The Duke Cardiovascular Research Center, Duke University Medical Center, Duke University, CaRL Building, 213 Research Drive, Durham, NC 27710 USA

**Keywords:** Sp3, Skeletal muscle, Cardiac muscle, MNase-seq, ChIP-seq, DNA, Transcription, Cardiovascular biology

## Abstract

In a previous report, we demonstrated that Cbx1, PurB and Sp3 inhibited cardiac muscle differentiation by increasing nucleosome density around cardiac muscle gene promoters. Since cardiac and skeletal muscle express many of the same proteins, we asked if Cbx1, PurB and Sp3 similarly regulated skeletal muscle differentiation. In a C2C12 model of skeletal muscle differentiation, Cbx1 and PurB knockdown increased myotube formation. In contrast, Sp3 knockdown inhibited myotube formation, suggesting that Sp3 played opposing roles in cardiac muscle and skeletal muscle differentiation. Consistent with this finding, Sp3 knockdown also inhibited various muscle-specific genes. The Cbx1, PurB and Sp3 proteins are believed to influence gene-expression in part by altering nucleosome position. Importantly, we developed a statistical approach to determine if changes in nucleosome positioning were significant and applied it to understanding the architecture of muscle-specific genes. Through this novel statistical approach, we found that during myogenic differentiation, skeletal muscle-specific genes undergo a set of unique nucleosome changes which differ significantly from those shown in commonly expressed muscle genes. While Sp3 binding was associated with nucleosome loss, there appeared no correlation with the aforementioned nucleosome changes. In summary, we have identified a novel role for Sp3 in skeletal muscle differentiation and through the application of quantifiable MNase-seq have discovered unique fingerprints of nucleosome changes for various classes of muscle genes during myogenic differentiation.

## Introduction

A thorough understanding of muscle differentiation is important for tissue regeneration. Cardiac and skeletal muscle differ in their regenerative capacity. While cardiac muscle cannot regenerate itself, skeletal muscle will fully regenerate if the injury is minor. However, if the injury is severe, skeletal muscle fails to fully regenerate. In the absence of complete healing, fibrotic tissue develops which impairs muscle function^1,2^. In a recent study, we showed that the reprogramming of scar tissue fibroblasts into cardiac muscle cells required the loss Cbx1, PurB and Sp3^3^. These three proteins functioned as a complex and inhibited cardiac muscle gene expression in-part by manipulating nucleosome positioning. In precursor cells, the Cbx1-PurB-Sp3 complex induced nucleosomes to tightly cluster on cardiac muscle genes. The tight clustering of nucleosomes prevented RNA-pol-II from binding^3^. Ablation of Cbx1, PurB or Sp3 was sufficient to induce cardiac muscle differentiation. Nucleosome structure on cardiac muscle genes relaxed, with increasing space between nucleosomes surrounding the transcription start site. The increased space between nucleosomes allowed RNA-pol-II to bind and initiate transcription^3,4^.

While cardiac and skeletal muscle share a number of similarities and express a number of common proteins^5^, there are substantial differences between the two types of muscle. Skeletal muscle cells are multinucleated and are long unbranched cylinders; in contrast, cardiac muscle cells tend to have a single nucleus and they form long fibers by attaching to each other. Differences are also seen in the proteins these cells express. The sarcomere, the structure that generates force in muscle, is comprised of more than twenty proteins and the majority are expressed in both skeletal and cardiac muscle. However, while Tnni3 is highly expressed in cardiac muscle it is virtually absent in skeletal muscle. In place of Tnni3, skeletal muscles express Tnni1 and Tnni2^6^. Similarly, while skeletal muscle expresses a full-length Nebulin protein, in cardiac muscle a truncated version is expressed and is known as Nebulette^7^. There are also differences in ion channel expression with cardiac muscle expressing Cacna1c, Ryr2 and Scn5a while skeletal muscles express Cacna1s, Ryr4 and Scn4a^8–10^. Considering the similarities and differences between skeletal and cardiac muscle, we were curious to determine if Cbx1, PurB and Sp3 regulated the differentiation of both muscle types or if the effects were specific for cardiac muscle.

We have previously demonstrated a role for Cbx1, PurB, and Sp3 in cardiac muscle differentiation using a combination of ChIP-seq and MNase-seq. The standard approach for ChIP-seq data analysis is to identify binding sites in the genome, ascribe these peaks to genes and interrogate the subsequent list of genes via gene ontological methods to infer biological meaning. Gene ontology is a controlled and structured vocabulary of terms that aims to provide a specific definition of protein functions. Essentially, each protein is ascribed to one or more biological functions and each biological function is given a unique gene ontology term. Significant biological insights have been made through the application of gene ontology; however, the approach is not without its pitfalls and various biases can lead to data misinterpretation^11^. In addition, the varying levels of detail within each gene ontology term can make it difficult to infer biological meaning. We have found this to be especially true with regards to studying cellular differentiation as gene ontology terms can contain genes corresponding to multiple lineages^4^. With respect to MNase-seq, the data is analyzed to infer nucleosome positioning. While algorithms have been designed to report changes in nucleosome architecture in a statistical manner^12,13^, typically they are reported in a descriptive manner.

In this study, we had two objectives. The first objective was to determine the role of Cbx1, PurB and Sp3 in skeletal muscle differentiation. Based on the data presented in this study, Cbx1 and PurB appear to negatively regulate both cardiac muscle and skeletal muscle differentiation. Interestingly, Sp3 appears to be necessary for skeletal muscle differentiation. The second objective of this study was to further develop our approach for the integration of ChIP-seq and MNase-seq datasets. In previous reports, we have described a modified ChIP-seq data analysis approach whereby gene ontology is replaced with groups of defined lineage-specific genes^3,4^. In this report, we further develop our approach by describing a method to determine if nucleosome repositioning is significant in specific groups of genes. Through this novel method, we discovered that skeletal muscle-specifc genes show a pattern of nucleosome changes that are distinct from both heart muscle-specific and commonly expressed muscle genes.

## Results

### Nucleosome dynamics in skeletal muscle cell differentiation

**Figure 1:**
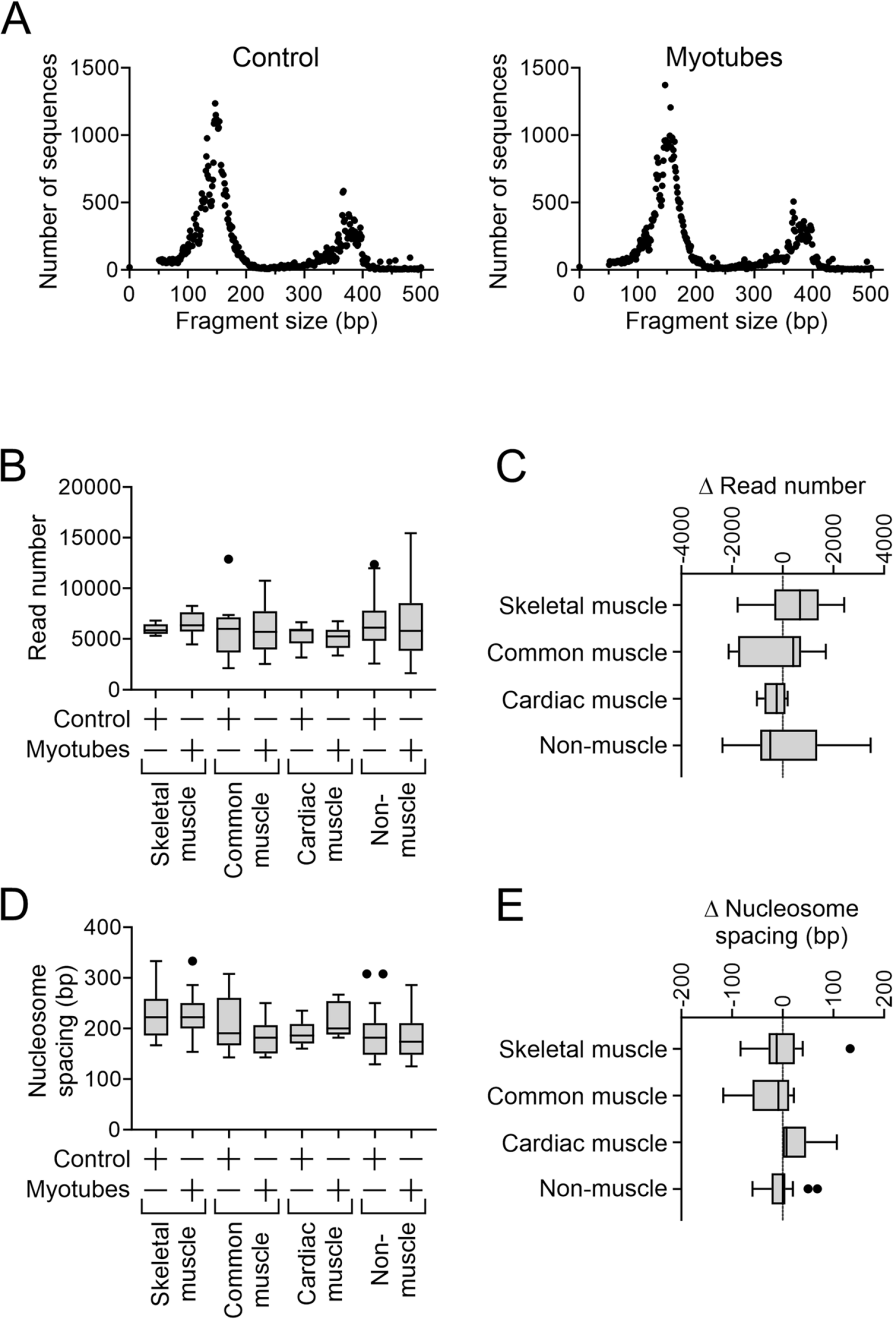
Skeletal muscle differentiation is associated with dynamic re-positioning of nucleosomes. Chromatin was isolated from C2C12 cells immediately prior to the start of skeletal muscle differentiation (control) and seven days later when myotubes were evident. Once isolated, the chromatin was digested with MNase and the resultant digests were sequenced. (**A**) Sequence size distributions after MNase digestion. (**B**) Sequences were aligned to the mouse genome and the number of sequences (read count) which aligned to various promoters (defined as − 3kb to + 1kb with respect to the transcription start site (TSS) were determined at a 1bp resolution. Defined groups of muscle-specific and non-muscle genes were analyzed. The muscle-specific group were split further on the basis of their expression: (1) muscle-specific genes expressed solely in skeletal muscle; (2) muscle-specific genes expressed in both skeletal and cardiac muscle; and (3) muscle-specific genes expressed solely in cardiac muscle. These groups are called skeletal muscle-specific, common and heart muscle-specific respectively. The number of genes in each group ranged from 6 to 20. The graph shows the summed read number over the full length of the promoter. No statistical differences (ANOVA) were observed. (**C**) For each analyzed promoter, the change in read number (Δ Read count) following myogenic differentiation was calculated. N = 6–20 per group. No statistical differences (ANOVA) were observed. (**D**) For each analyzed promoter, the nucleosome number was determined. The graph shows average nucleosome spacing. Average nucleosome spacing was determined by dividing the promoter length (4kb) by the number of nucleosomes. N = 6–20 per group. No statistical differences (ANOVA) were observed. (**E**) For each analyzed promoter, the change in average nucleosome spacing (Δ Nucleosome spacing) following skeletal muscle differentiation was determined. The change in average nucleosome spacing was determined by subtracting average nucleosome spacing in control cells from the average nucleosome spacing observed in myotubes. N = 6–20 per group. No statistical differences (ANOVA) were observed.

The first objective was to determine nucleosome changes in cells undergoing skeletal muscle differentiation. To that end, chromatin from undifferentiated C2C12 cells and C2C12-derived myotubes was digested with MNase to liberate nucleosomes. Since MNase digestion can be aggressive, we titrated the enzyme to give rise to a mixture of mono- and di-nucleosomes^13,17^ (Fig. 1A) thus retaining the maximum amount of information.

A large number of genes are known to be muscle-specific. Several muscle-specific genes like Actn2 and Ttn are expressed in both skeletal muscle and cardiac muscle. However, a number of muscle-specific genes show a more restricted pattern of expression. Expression of the muscle-specific genes Neb, Tnni1 and Tnni2 is restricted to skeletal muscle. Similarly, Tnni3 and Nebl are only found in cardiac muscle. To reflect this, we analyzed and compared three groups of muscle-specific genes: (1) expressed solely in skeletal muscle; (2) expressed in both skeletal muscle and cardiac muscle; and (3) expressed solely in cardiac muscle. By way of a control, we also analyzed a fourth group comprised of non-muscle genes.

Promoters, defined as being − 3kb to + 1kp of the transcription start site, were evaluated for their nucleosome content and architecture. Nucleosome content did not appreciably differ between the different groups of gene promoters (Fig. 1B). Moreover, there was no significant change in nucleosome content in any group during skeletal muscle differentiation (Fig. 1C). A general overview of nucleosome architecture was determined by calculating the average nucleosome spacing over the 4kb region. Again, there was no significant change in average nucleosome spacing between the four groups of gene promoters (Fig. 1D) and the average nucleosome spacing did not change with skeletal muscle differentiation (Fig. 1E).

**Figure 2:**
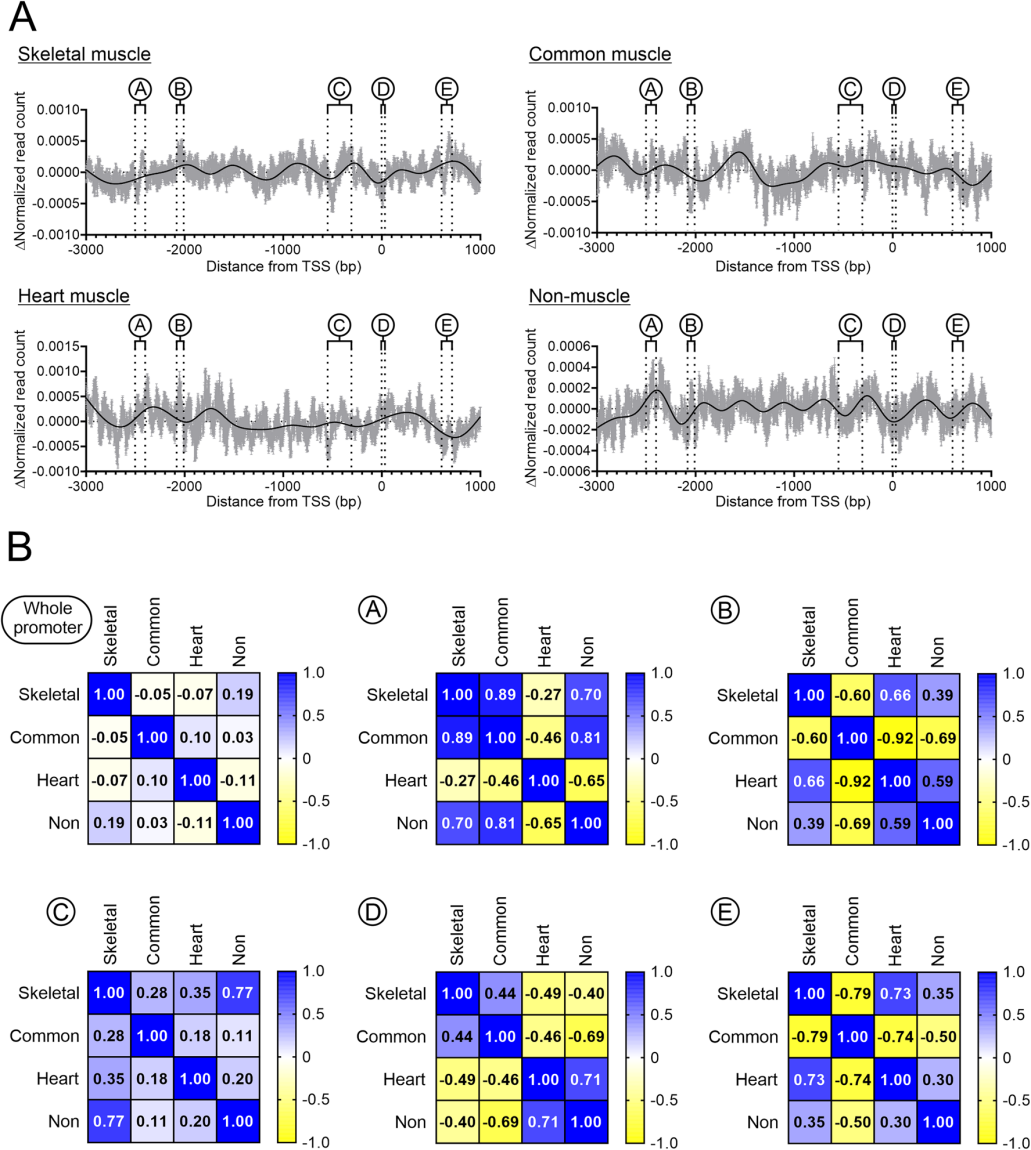
Quantifying MNase-seq to determine changes in nucleosome positioning during skeletal muscle differentiation. (**A**) To compare nucleosome patterns within each group and across the four groups, the read counts were normalized. Normalization was carried out dividing the read count at each base-pair of the promoter by the sum of read counts across the promoter (a full description is provided in the methods) and was carried out to ensure that each promoter had equal weight. The graphs show the change in normalized read count for each group (skeletal muscle-specific, common muscle, heart muscle-specific, and non-muscle genes) following myogenic differentiation. The light grey bars show the mean and standard error a 1bp resolution. The black line represents the best-fit model of the data. Statistical analysis of the skeletal muscle-specific group identified five regions that were significantly different in control cells and myotubes. These regions are labelled A–E. (**B**) for regions A–E, correlations were determined between the four groups.

The lack of any appreciable loss of nucleosome content suggested that during skeletal muscle differentiation, nucleosomes were not lost but were either static or were repositioned relative to each other. To investigate nucleosome architecture in more detail, we developed an algorithm to evaluate nucleosome content changes in undifferentiated C2C12s and skeletal myotubes at a base-pair resolution. Through this approach, we discovered five regions in skeletal muscle-specific gene promoters that differed significantly in their nucleosome content between skeletal myotubes and their undifferentiated precursors (Fig. 2A). With respect to the transcription start site, these five regions were located throughout the skeletal muscle-specific promoters: − 2500bp to − 2400bp, − 2100bp to − 2000bp, − 650bp to − 400bp, + 5bp to + 20bp, and + 600bp to + 700bp. Henceforth, these regions will be referred to as A, B, C, D, and E respectively. In skeletal muscle-specific gene promoters, regions A, C and E share a similar pattern indicating that they are nucleosomes moving towards the 3’ end of the gene. In contrast, region B is a region of nucleosome build-up and region D is a region of nucleosome loss (Fig. 2A).

To compare regions A–E in the four groups of genes, Pearson’s R-values were used. An R-value greater than 0.7 is commonly regarded as indicative of a strong correlation^18^. Over the whole promoter region, there was no correlation between the four groups with respect to changes in nucleosome content (Fig. 2B). In contrast, strong correlations were noted in Regions A–E. As mentioned above, regions A, C, and E were regions where nucleosomes were moving downstream in skeletal muscle-specific gene promoters. Nucleosome movement downstream was also observed in regions A and C of non-muscle gene promoters (Fig. 2B). No nucleosome movement was observed in region E of non-muscle gene promoters. While the nucleosome in region E showed the same movement downstream in heart muscle-specific genes, the nucleosome in common muscle genes moved in the opposite direction (Fig. 2B). The same situation was found in region B. While heart muscle-specific genes showed a similar nucleosome build-up as skeletal muscle-specific genes; common muscle genes lost nucleosome content at this position (Fig. 2B). Region D surrounded the transcription start-site. Common muscle genes showed the most similarity to skeletal muscle-specific genes, while heart muscle-specific genes and non-muscle genes showed a tendency towards the opposite with nucleosome build-up (Fig. 2B). While common muscle genes were most similar to skeletal muscle-specific genes, it should be noted that nucleosome loss was only significant for the latter group (Supplementary Fig. 1).

### Sp3 is a positive regulator of skeletal muscle differentiation

In a previous study, we demonstrated that cardiac muscle differentiation required the loss of three transcription repressors: Cbx1, PurB and Sp3^3^. These repressors regulated cardiac muscle differentiation via nucleosome positioning^3^. Consequently, we wanted to determine what role, if any, these proteins played in skeletal muscle differentiation and whether they influenced the changes in nucleosome positioning that we had observed.

**Figure 3:**
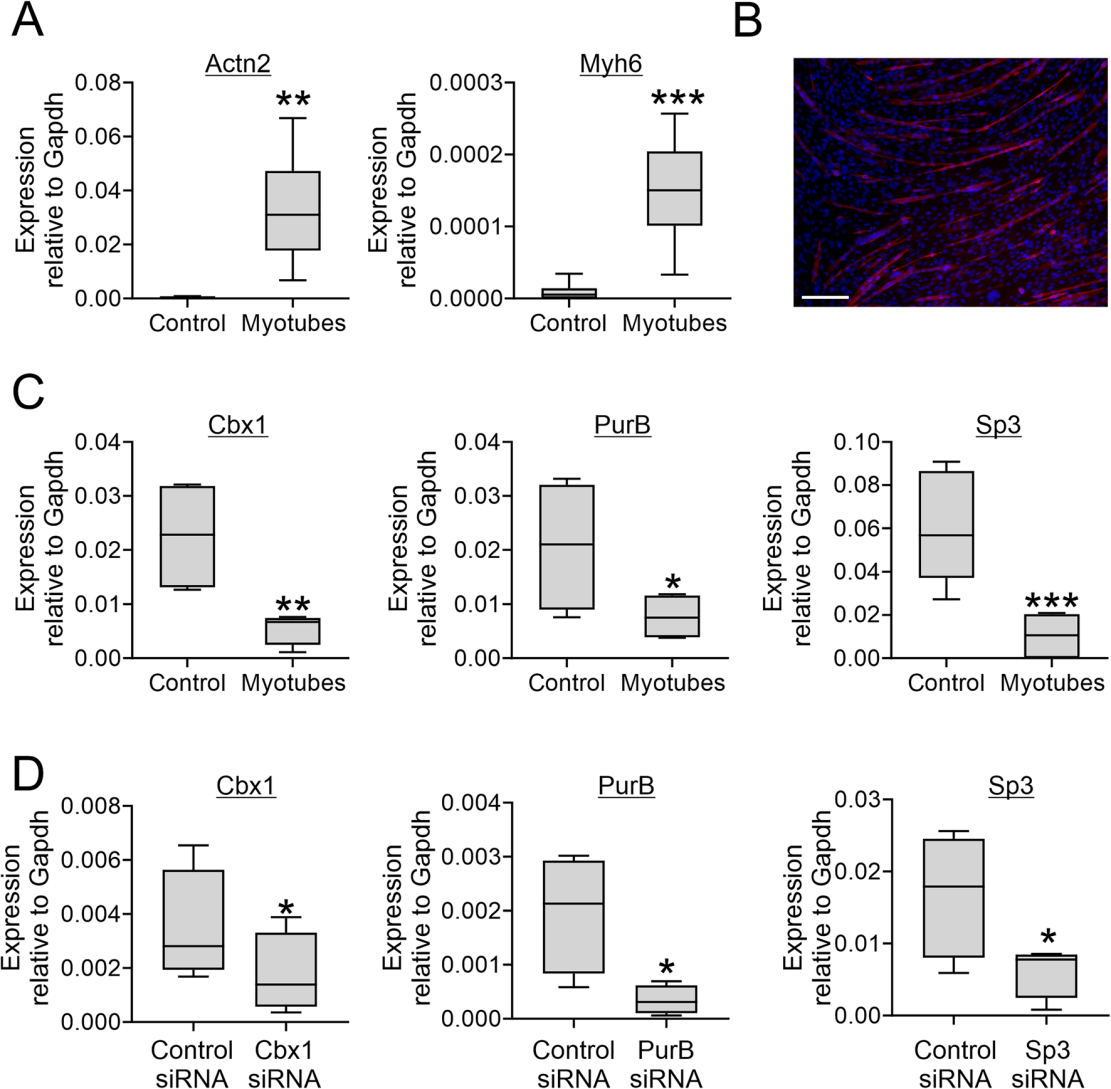
Cbx1, PurB and Sp3 play opposing roles on myotube formation. (**A**–**C**) C2C12 cells were grown to confluence whereupon they underwent differentiation to generate myotubes. (**A**) RNA was isolated immediately prior to differentiation (control) and after 6 days of skeletal muscle differentiation. Expression of the muscle-specific genes Actn2 and Myh6 was determined by qPCR and expression levels relative to the housekeeping gene Gapdh are shown. N = 6. Independent *T*-test, **P < 0.01, ***P < 0.001. (**B**) Myotubes were visualized by immunostaining for the muscle-specific protein Actn2. Nuclei were counterstained with DAPI. Scale bar 200 microns. The image is representative of three independent experiments. (**C**) RNA was isolated immediately prior to differentiation (control) and after 8 days of skeletal muscle differentiation. Expression of Cbx1, PurB and Sp3 was determined by qPCR and expression levels relative to the housekeeping gene Gapdh are shown. N = 4. Independent T-test, *P < 0.05, **P < 0.01, ***P < 0.001. (**D**) C2C12 cells were transfected with siRNAs targeting Cbx1, PurB, or Sp3. A non-targeting siRNA was used as a control. Three days later, cells were analyzed for RNA levels. Expression of Cbx1, PurB and Sp3 was determined by qPCR and expression levels relative to the housekeeping gene Gapdh are shown. N = 4. Independent *T*-test, *P < 0.05, **P < 0.01, ***P < 0.001.

Skeletal muscle differentiation in C2C12 cells was robust with significant increases in the expression of muscle-specific genes (Fig. 3A) and significant myotube formation (Fig. 3B). Akin to cardiac muscle differentiation, skeletal muscle differentiation was associated with a significant loss of Cbx1, PurB and Sp3 RNA (Fig. 3C). Repressor expression was targeted by siRNA prior to the start of differentiation and inhibition of expression at the transcript level was robust (Fig. 3D).

**Figure 4:**
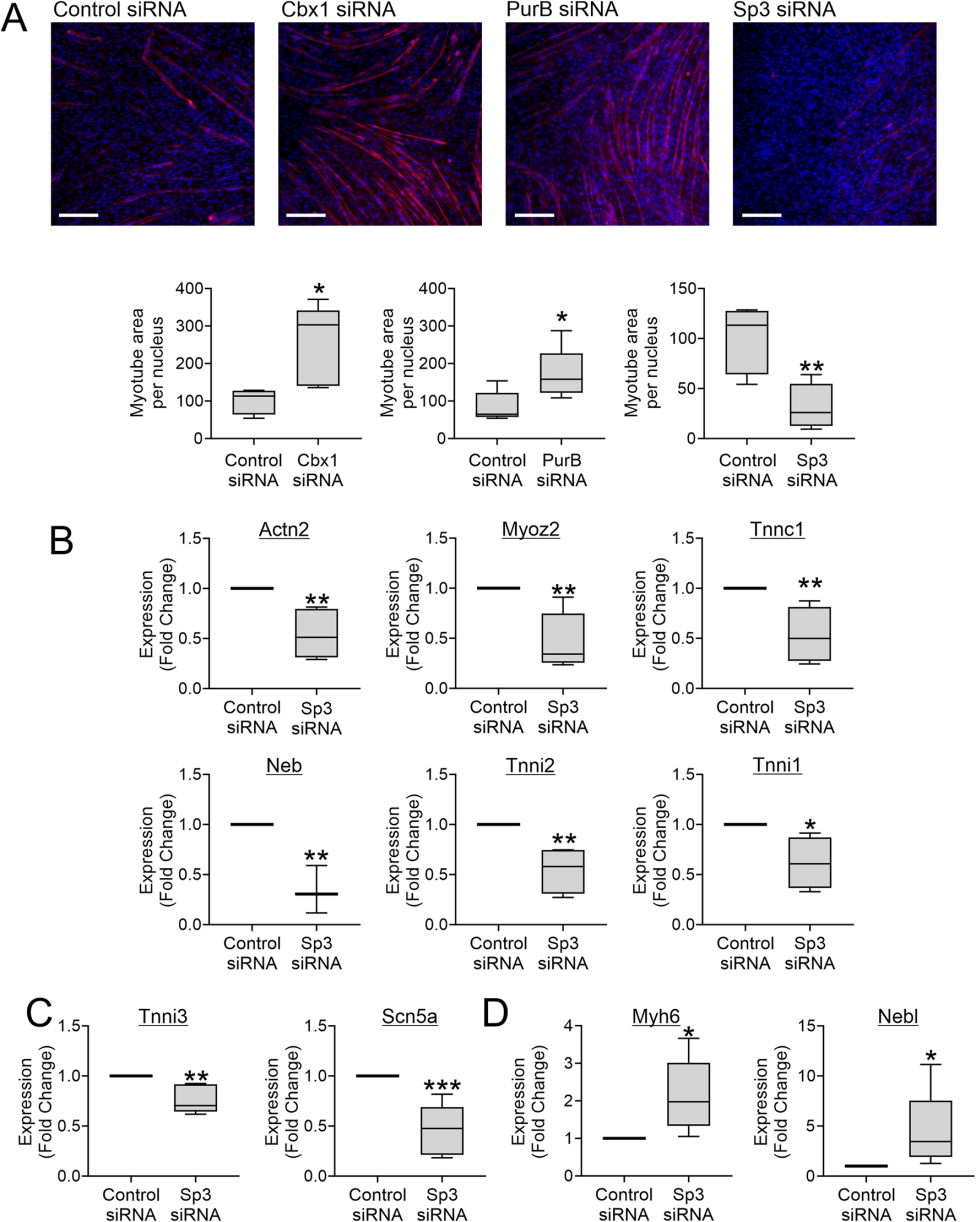
Sp3 plays opposing roles on muscle gene expression. (**A**), Three days prior to the start of skeletal muscle differentiation, C2C12 cells were transfected with siRNAs targeting Cbx1, PurB, or Sp3. A non-targeting siRNA was used as a control. Skeletal muscle differentiation was assessed by immunostaining cells with the muscle-specific protein Actn2. Images were taken at five random locations. Both myotube area and nuclei number were counted. Skeletal muscle differentiation is expressed as the myotube area per nucleus. Representative images (scale bar 200 microns) and quantification are shown from one of three independent experiments. Independent T-test, *P < 0.05, **P < 0.01. (**B–D**) C2C12 cells were underwent skeletal muscle differentiation for seven days. RNA was extracted and analyzed for the indicated muscle-specific mRNAs by qPCR. Gapdh was used as a housekeeping control. Expression is shown relative to cells transfected with non-targeting siRNA. (**B**) Skeletal muscle-specific and common muscle gene expression, **C** heart muscle-specific gene expression, (**D**) muscle genes up-regulated by Sp3 knockdown. N = 4–5. Independent *T*-test, *P < 0.05, **P < 0.01, ***P < 0.001.

Targeted knockdown of Cbx1 or PurB prior to the start of myogenic differentiation increased the ability of C2C12 cells to generate myotubes (Fig. 4A). In contrast, Sp3 knockdown inhibited myotube formation. The result of Sp3 knockdown on myotube formation was unexpected and intriguing, thus we investigated the effect of Sp3 in more detail and focused on muscle-specific gene expression. Muscle genes were chosen from the skeletal muscle-specific, common and heart muscle-specific groups. As expected, these genes had a wide degree of expression (Supplementary Fig. 2). Sp3 knockdown was found to inhibit the expression of genes from all three groups (Fig. 4B and Fig. 4C). In contrast, Sp3 knockdown increased the expression of Myh6 and Nebl (Fig. 4D).

**Figure 5:**
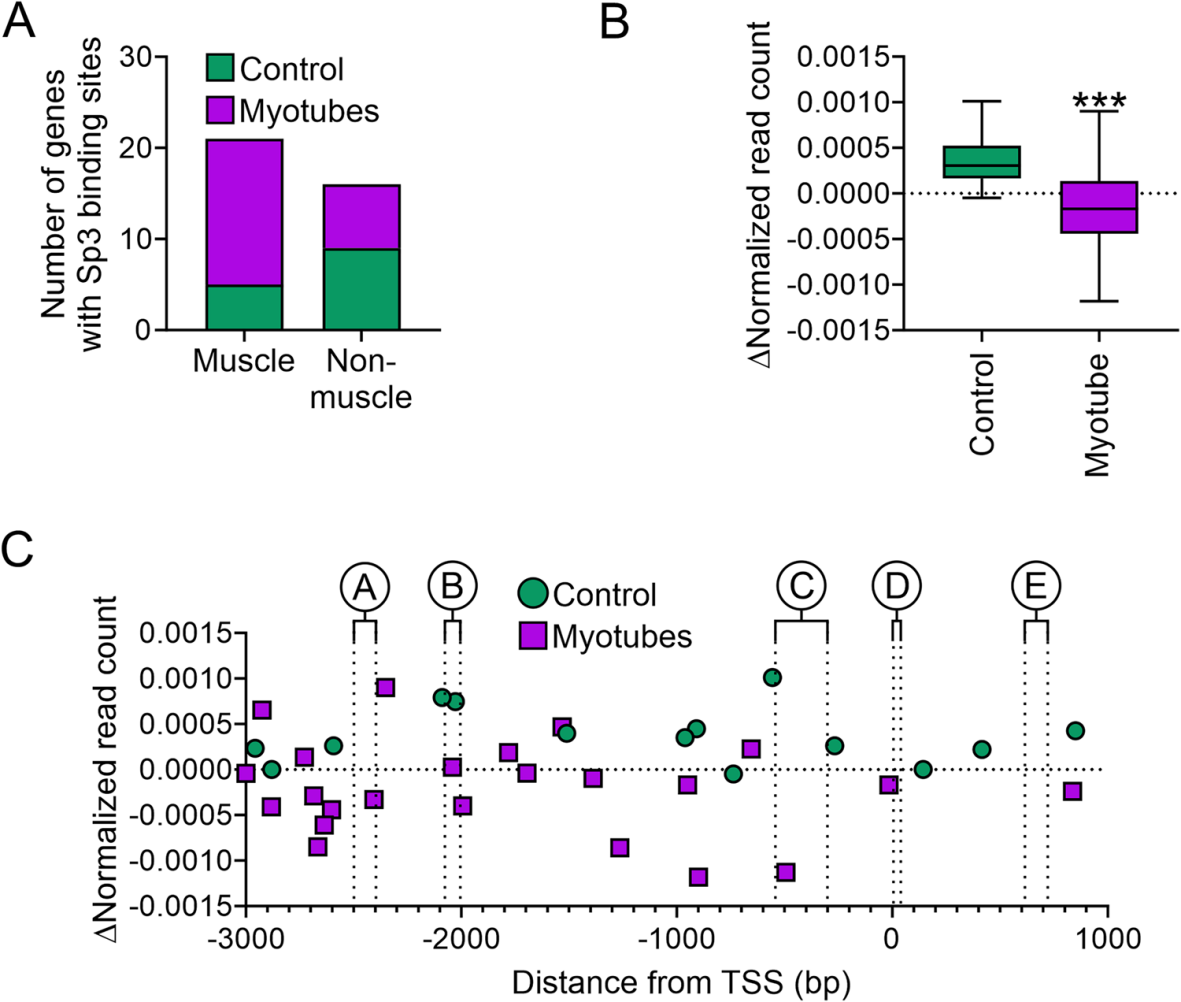
Sp3 binding in muscle genes. (**A**) ChIP-seq was used to evaluate Sp3 binding in C2C12 cells immediately prior to differentiation (control) and after seven days of skeletal muscle differentiation (myotubes). The graph shows the number of muscle-specific and non-muscle genes with a validated Sp3 binding site. (**B**) For each Sp3 binding site, the change in nucleosome content following myogenic differentiation was calculated. The two bars represent Sp3 binding sites found only in non-differentiated and differentiated cells, respectively. (**C**) Sp3 binding sites (muscle and non-muscle) are shown with respect to the TSS and regions A–E identified in skeletal muscle-specific gene promoters. Sequences surrounding the binding peak are shown in Supplementary Table 1.

ChIP-seq was used to identify Sp3 binding in the genome. Sp3 was found to bind to both muscle and non-muscle genes. However, there was a difference in where the majority of Sp3 was localized in control cells and myotubes. In control cells, Sp3 was bound mostly to non-muscle genes. In myotubes, Sp3 was bound predominantly to skeletal and common muscle genes (Fig. 5A). None of the Sp3 binding sites observed in control cells were retained differentiation. During differentiation, Sp3 binding sites in control cells showed an increase in nucleosome content (Fig. 5B). In contrast, Sp3 binding sites in myotubes were associated with a significant loss in nucleosome content (Fig. 5B). While there was a loss of nucleosome content, they generally did not align with regions A–E (Fig. 5C). Curiously, the majority of Sp3 binding sites were found 2000 to 3000 bp upstream of the transcription start site (Fig. 5C).

## Discussion

In a previous study, we modified the standard ChIP-seq analysis approach to investigate the effects of innate immunity on cellular reprogramming^4^. The standard ChIP-seq analysis approach involves deriving biological relevance through gene ontology. Binding sites are identified in the genome and assigned to genes. The resultant list of genes is subsequently matched to a set of gene ontological terms. Gene ontological terms are curated lists of genes believed to have the same function. Gene ontology enrichment, gene ontology terms that appear more frequently than would be expected by chance, is used to infer biological meaning. Due to their curated nature, gene ontological terms do not always map well onto the research question that is being asked^11^. This has been the case in our research. A major focus of our laboratory is the reprogramming of fibroblasts into cardiac muscle cells and it is important to determine if reprogramming cells adopt characteristics of alternative lineages, notably endothelial and neuronal. Unfortunately, we have found that we cannot fully assess reprogramming trajectories through gene ontology. This is due to a mismatch between the constituent genes of gene ontological terms and the genes whose expression we need to measure. To that end, we developed an alternative approach whereby gene ontology terms were replaced by genes known to be specifically expressed in cardiac muscle cells, endothelial cells, neurons and fibroblasts. Through this approach, we were able to fully map the impact of a Rig1-YY1 pathway on both the efficacy of reprogramming as well as the specificity of reprogramming towards the cardiac muscle lineage versus alternative lineages^4^. For similar reasons, we have also had to modify the standard approaches to analyzing MNase-seq data. MNase-seq is used to assess nucleosome positions and this is important in the context of reprogramming because the initial stages are epigenetic in nature^24^. Various computer programs have been developed to analyze MNase-seq data^15,25,26^. The motivation of their designers was to determine if changes in nucleosome positioning were significant. Despite this, the literature abounds with characterizations of nucleosome positioning that are solely descriptive. In this manuscript, we report a novel statistical method for determining if changes in nucleosome positioning are significant. We again applied it to looking at specific classes of genes. In this report, we looked at muscle and non-muscle genes. The muscle genes were further stratified into those whose pattern of expression which was common to both heart and skeletal muscle, skeletal muscle-specific, and heart muscle-specific. Through this approach, we determined that skeletal muscle-specific genes undergo significant nucleosome repositioning at five locations in the promoter region. Three of these regions, where nucleosome depletion was followed by nucleosome buildup, were the result of nucleosome movement towards the 3’end of the gene. Nucleosome movement in these regions was not unique to skeletal muscle-specific genes as the same was observed in both non-muscle and heart muscle-specific genes. Similarly, nucleosome build-up in a region 2000 to 2100bp upstream of the transcription start site was found in both skeletal muscle-specific and heart muscle-specific promoters. Only one significant region appears to specifically mark skeletal muscle-specific promoters and that was the significant loss of nucleosomes immediately downstream of the transcription start site. This is a previously reported feature of skeletal muscle differentiation and is believed to be necessary for RNA-pol-II binding to the promoter^27^. In this context, it is interesting that heart muscle-specific genes were found to undergo significant nucleosome build-up in this region. Nucleosome build-up would act to inhibit RNA-pol-II and may explain how precursors to commit to either skeletal or heart lineage. However, muscle genes that are expressed in both skeletal and cardiac muscles did not undergo any loss of nucleosomes around the transcription start site. This may indicate that cells regulate restricted and non-restricted muscle genes differently. In support, we found that nucleosome changes in regions B and E were strongly negatively correlated in skeletal muscle-specific and commonly expressed muscle genes. Alternatively, it may indicate that nucleosome loss in this region is not fundamental to gene expression. One caveat with our approach is that the results may be dependent on the number of genes analyzed.

In cardiac muscle differentiation, Cbx1, PurB and Sp3 are inhibitors^3,4^. Cardiac and skeletal muscles share many of the same proteins, and thus we hypothesized that Cbx1, PurB and Sp3 would similarly inhibit skeletal muscle differentiation. In support of the hypothesis, expression of all three proteins was significantly reduced during skeletal muscle differentiation. Considering that skeletal muscle differentiation reduced their expression, we targeted Cbx1, PurB and Sp3 using siRNAs prior to differentiation. Targeting an inhibitor at this stage would be expected to enhance differentiation^3,4^. Indeed, knockdown of either Cbx1 or PurB was sufficient to enhance the ability of C2C12 cells to differentiate into myotubes. Thus, Cbx1 and PurB appear to be general inhibitors of muscle differentiation in that they inhibit both cardiac and skeletal muscle differentiation. Sp3 provided a more interesting case as knockdown of this protein prior to differentiation decreased myotube number. Further evidence for a positive role in skeletal muscle differentiation came from analysis of muscle-specific genes, where Sp3 knockdown was shown to inhibit the expression of Actn2, Myoz2, Neb, Ryr2, Tnnc1, Tnni1, and Tnni2. This is in agreement with an earlier study which demonstrated that Sp3 inhibits Myh7 expression in inactive skeletal muscles^19^. However, we also found that Sp3 negatively regulated the expression of two muscle specific genes, Myh6 and Nebl. Sp3 is known to have opposing effects on different promoters within the same cell-type^20–23^. The different effects on muscle genes may be a consequence of the significantly lower basal expression of Myh6 and Nebl versus the other proteins or may be related to differences in the promoters^20–23^. In cardiac muscle precursors, Sp3 resides in a complex with Cbx1 and PurB^3^. The implication is that in skeletal muscle precursors, Sp3 is binding with greater affinity to another protein (or proteins) which enables Sp3 to change from an inhibitor to an inducer of differentiation. The ability of Sp3 to change from an inhibitor to an inducer may explain how precursors differentiate into skeletal muscle versus cardiac muscle. Sp3 binding was associated with a decrease of nucleosome content. This would be expected if Sp3 was acting as a pioneer transcription factor. However, due to low correlation, it is unlikely that Sp3 mediates the aforementioned changes in nucleosome patterning that is seen in skeletal muscle-specific genes during myoblast differentiation.

As mentioned earlier, muscles often need support to fully regenerate^1,2^. Cardiac muscle lacks any capacity to regenerate and many methods for muscle restoration are being investigated. We have been investigating the reprogramming of scar tissue fibroblasts into cardiac muscle. However, cellular reprogramming is an inherently low efficacy approach due to a number of barriers. We originally identified Cbx1, PurB and Sp3 as one such barrier; deletion of the genes was found to improve reprogramming efficacy. Skeletal muscle has a higher capacity to regenerate. However, regeneration is often incomplete after significant injuries. It was natural to assume that targeting Cbx1, PurB and Sp3 would enhance the regenerative capacity of skeletal muscle precursors. However, while our data does suggest targeting Cbx1 and PurB would be useful in this regard, targeting Sp3 would likely be deleterious. Our data also suggests another approach for improving the efficacy of skeletal muscle regeneration and that would be targeting nucleosomes surrounding the transcription start site. In our analysis of nucleosome structural changes, skeletal muscle-specific genes differed markedly from commonly expressed and heart muscle-specific genes in that they showed loss of nucleosomes immediately surrounding the transcription start site. Seeing that this appears to be a unique marker of skeletal muscle genes, further nucleosome loss through a modified gene-editing technology could further enhance their expression.

In summary, we have developed a novel method to analyze nucleosome architecture and deployed it to understand differences between skeletal muscle-specific, heart muscle-specific and non-restricted muscle genes. In addition, we have identified Sp3 as a novel player in skeletal muscle differentiation and have highlighted key changes in nucleosome architecture in this process. Further work will be necessary to confirm these findings in vivo.

## Materials and methods

### Skeletal muscle differentiation

C2C12 cells (passage 5–10) were maintained in growth media containing DMEM supplemented with 15%v/v FBS (Thermo Scientific Hyclone Fetal bovine serum, Catalogue number SH30071.03, Lot number AXK49952) and 1%v/v penicillin/streptomycin (Gibco, Catalogue number 15140-122, 100units Penicillin, 100ug/ml Streptomycin). The cells were acquired from ATCC (Catalogue number 30–2002). Cells were routinely passaged once the monolayer had reached 70–80% confluence using 0.05% w/v trypsin (Gibco, Catalogue number 25300–054)^3^. For differentiation, C2C12 cells were seeded at 75,000 cells/cm^2^ in growth media. The cells were allowed to reach confluence (day 0 of the differentiation protocol) and skeletal muscle differentiation was carried out by culturing the cells in differentiation media (DMEM, 10%v/v FBS). Media was refreshed every 48 h. Differentiation was assessed seven days after cell seeding.

### siRNA knockdown

Cbx1, PurB and Sp3 targeting siRNAs have been described previously^3^. The siRNAs were made to 20 μM in nuclease free water and stored at − 80 °C until use. Transfection occurred one day after C2C12 seeding (day -3 of the differentiation protocol). For each well, 0.75 μl of the working siRNA solution was diluted with 99.25 μl Optimem‐Serum Free media. In a separate tube, 0.75 μl of Dharmafect‐I (Dharmacon) was diluted with 99.25 μl Optimem‐Serum Free media. After 5 min incubation, the two solutions were combined. After 20 min, the transfection complexes and 550 μl growth medium were added to the cells.

### qPCR

Total RNA was extracted using Quick-RNA MiniPrep Kit according to the manufacturer’s instructions (Zymo Research). Total RNA (50ng-100ng) was converted to cDNA using a high capacity cDNA reverse transcription kit (Applied Biosystems). cDNA was used in a standard qPCR reaction involving FAM conjugated gene specific primers (ThermoFisher) and TaqMan Gene Expression Master Mix (ThermoFisher). Primers were acquired from ThermoFisher and the assay ID numbers are: Actn2 Mm00473657_m1; Cacna1c Mm00437917_m1; Gapdh Mm99999915_m1; Kcna4 Mm01336166_m1; Kcnh2 Mm00465377_mH; Kcnj2 Mm00434616_m1; Myh6 Mm00440359_m1; Myoz2 Mm00469639_m1; Neb Mm01546298_m1; Nebl Mm00503886_m1; Ryr1 Mm01175211_m1; Ryr2 Mm00465877_m1; Scn4a Mm00500103_m1; Scn5a Mm01342518_m1; Tnnc1 Mm00437111_m1; Tnni1 Mm00502426_m1; Tnni2 Mm00437157_g1; Tnni3 Mm00437164_m1; Tnnt2 Mm01290256_m1; Ttn Mm00621005_m1^3^.

### Immunostaining

Myotubes were visualized by immunostaining for the muscle protein Actn2 as previously described^14^. In brief, cells were fixed with 4% PFA incubated overnight at 4°C with Actn2 antibody (1:50 in antibody buffer (1xPBS, 0.1% Tween-20, 5% BSA)). Cells were then washed three times in antibody buffer (5 min per wash, room temperature) and incubated for a further hour with a 1:100 dilution of an Alexa-Fluor 594 antibody (ThermoFisher, Cat no A21201) in antibody buffer at room temperature. In the last 30 min of the incubation, DAPI was added to a final concentration of 1µg/ml to stain nuclei. Following washing in PBS to remove unbound complexes, immunofluorescence was measured using a Zeiss Axiovert 200 inverted microscope. Areas occupied by Actn2 and DAPI staining were determined by Image J^3^.

### MNase-seq

Chromatin was isolated from cells at day 0 (control) and day 7 (myotubes) via a SimpleChIP® Plus Enzymatic Chromatin IP Kit (Cell Signaling #9005) according to the manufacturer’s instructions. MNase was added at the optimized dose (6µl of a 1:10 dilution, 20 min, 37°C). The amount of MNase was empirically determined to minimally digest the chromatin whereby dinucleosomes were almost fully digested (> 95%) to mononucleosomes. High-throughput sequencing was performed by the Duke Genomic Core. In total, two independent experiments were performed and libraries generated with a NovaSeq 6000 kit (Illumina). Libraries were pooled and run in duplicate (50bp paired-end) with an Illumina NovaSeq 6000. Sequencing depth was > 25 × 10^6^ individual reads per sample. Individual bioinformatics programs within the Galaxy suite were used for sequence alignment etc.^3^. Sequences were aligned to mouse reference genome mm10 using Bowtie2. The aligned sequences were then filtered for quality (phred scale > 30), true pairs, and for the removal of mitochondrial genome sequences. The sequence size was calculated via the Paired-end histogram of insert size frequency function in Galaxy^3^.

At this point, our approach diverges from those that have been previously published^15,16^. Those prior approaches identify peaks (nucleosomes) either by smoothing the data through fourier analysis or by viewing the data set as a sum of Poisson distributions. Our approach does not smooth the data nor model the data onto an idealized structure. Instead, the BamCoverage algorithm was used to determine read count for each promoter (-3kb upstream of the transcription start site to + 1kb downstream of the transcription start site) for the genes listed below. The parameters used for the BamCoverage algorithm were: (1) a window size of 1bp; (2) normalizing coverage to the effective mouse genome size (mm10); (3) Mnase-seq mode where only the 3 nucleotides at the center of each fragment are counted (the fragment ends are defined by the two mate reads and only fragments between 130 and 200 bases were used). The data was exported in bedgraph format. The bedgraph format does not retain the original 1bp window size (regions of identical read coverage are listed as a range, from starting to end position) and the BamCoverage algorithm reports each sample in a separate file. To restore the 1bp window size and to compare each sample together, the various files were combined via the bedtools Merge BedGraph files algorithm. The bedtools Merge BedGraph files algorithm was also used to compare (align) various genes. Here, the bedgraph files were re-annotated by replacing the original chromosome location with an arbitrary chromosome (ChrX) and the genomic locations re-ordered to match their position relative to the transcription start site, noting that genes can be in either a plus or minus orientation.

An important part of this study is a comparison of a priori defined groups of genes. Analyzed genes were broken down into four groups:

- Group 1 genes—muscle genes expressed solely in skeletal muscle: Neb, Scn4a, Cacna1s, Tnni1, Tnni2, Myh1, Myh2, Myh3, Myh4, Myogenin, Myod1, Ryr1.
- Group 2 genes—muscle genes expressed in both skeletal and cardiac muscle: Actn2, Kcnj2, Mef2C, Myh6, Myl2, Sln, Ttn, Myoglobin, Myh7.
- Group 3 genes—muscle genes expressed solely in cardiac muscle: Cacna1c, Kcna4, Nebl, Ryr2, Scn5a, and Tnni3.
- Group 4 genes—non-muscle genes: The non-muscle group comprised 26 genes that are specific to fibroblasts (Col1a2, S100a4, Thy1, Tcf21, Atl1, Col1a1, Postn), endothelial cells (Cd31, Cdh1, Eng, Flt1, Flt4, Pecam1, Tek, Vcam1, Vegfa, Vwf) and neurons (Dcx, Eno2, L1cam, Map2, Mapt, Ncam1, Neurod1, Nlgn1, Rbfox3, Syn1).

To ensure that the read counts of each gene were equally weighted, the read count data was normalized. This removed any bias from genes with significantly high or low read counts. Normalization was made to the sum of read counts according to the following formula:

For *gene x*, normalized read count_*position-n*_ = read count_*position-n*_/∑read count_*gene x*_ where *position-n* is the position of the 1bp window with respect to the transcription start site.

To determine the effects of skeletal muscle differentiation, a Δnormalized read count was calculated according to the following formula:

For *gene x*, Δnormalized read count_*position-n*_ = (read count_*position-n*_)_*myotube*_– (read count_*position-n*_)_*control*_.

Significances in Δnormalized read counts were determined by paired T-tests comparing normalized read counts in the control and myotube groups for each 1bp window.

Regression was applied to the datasets to generate the fitted curves. For spaces containing two peaks, regression was performed via the sum of two Lorentzians. For spaces with more than two peaks, regression was performed via a constrained spline. Fourier transforms indicated a dominant frequency of 1bp (the observation window) and thus were not suitable for regression.

### ChIP-seq

MNase digested chromatin was also used for ChIP-seq. MNase digested chromatin (~ 10 million cells) was incubated for 6 h at 4°C with 8µg Sp3 (ThermoFisher #PA5-78,176) antibody^3^. High-throughput sequencing was performed by the Duke Genomic Core. In total, two independent experiments were performed and libraries generated with a NovaSeq 6000 kit (Illumina). Libraries were pooled and run in duplicate (50bp paired-end) with an Illumina NovaSeq 6000. Sequencing depth was > 25 × 10^6^ individual reads per sample. Individual bioinformatics programs within the Galaxy suite were used for sequence alignment; peak calling; and peak comparisons. Adaptors were removed and sequences were then aligned to mouse reference genome mm10 using Bowtie2^3^. Read counts were determined for the above genomic regions and normalized as described above. Typical ChIP-seq peak callers such as MACS2 are designed to detect transcription factors (narrow peaks) and often fail to detect broad peaks which are the hallmark of chromatin binding proteins. We found that Sp3 is a chromatin binder^3^ and thus Sp3 binding is unlikely to be detected by typical ChIP-seq peak callers. Consequently, an alternative approach was adopted.

For each gene, the first step was to subtract the normalized read count of the input from the normalized read count of the Sp3 antibody pulldown. This is necessary to ensure that any peaks were the result of Sp3 binding and not the underlying chromatin. A Sp3 binding value was calculated for each 1bp window thus:

Sp3 binding value_*position-n*_ = (normalized read count_*position-n*_)_*Sp3 IP*_ – (normalized read count_*position-n*_)_*Input*_.

Peaks were deemed significant if they reached 4σ. To determine 4σ, the mean and standard deviation of the sum of Sp3 binding values across the full genomic window was calculated. A peak reached 4σ if the Sp3 binding value_*position-n*_ was greater than the mean + 4 standard deviations. A 4σ result has a confidence of 99.8%.

### Images

Images were prepared with CorelDraw and Zeiss software (Axiovision Rel4.8 and Zen Blue)^3^.

### Statistics

All statistical analysis was performed using Excel and GraphPad. Two tail T-Tests (2 groups) and one way ANOVAs (> 2 groups) were used as appropriate. For ANOVA, Bonferroni post-hoc tests were used to determine significance between groups^3^. Tukey Box-whisker plots are used. The plot shows distribution of data into quartiles, highlighting the mean (represented by a horizontal line), and outliers (represented by circles). The whiskers indicate variability outside the upper and lower quartiles, and any point outside those lines or whiskers is considered an outlier. For all statistical tests, a P-value of less than 0.05 was considered significant^3^.

### Supplementary Information

Supplementary Information.

## Data Availability

All of the data is presented in this study. Genomic data is available from the NIH Single Read Archive under accession number PRJNA1081841.
