# Supplementary material for: Skeletal muscle differentiation induces wide-ranging nucleosome repositioning in muscle gene promoters
Source: Sci Rep. 2024 Apr 24;14:9396. doi: 10.1038/s41598-024-60236-x (PMC11043329; doi:10.1038/s41598-024-60236-x)
Supplement: Supplementary file 1 — Supplementary Information. [file 41598_2024_60236_MOESM1_ESM.pdf]

Supplementary Material

Skeletal muscle differentiation induces wide-ranging nucleosome repositioning in muscle gene promoters: role for Sp3

Sonalí Harris[1], Iqra Anwar[1], Syeda S. Baksh[1], Richard E. Pratt[1], Victor J. Dzau[1], Conrad P. Hodgkinson[1,*]

Supplementary Figure 1. Analysis of nucleosome content surrounding the transcription start site. Region D is shown in more detail for each of the four groups analyzed. Regions significantly different between undifferentiated and differentiated cells are highlighted.

Supplementary Figure 2. Expression of skeletal muscle-specific, heart muscle-specific, and common muscle genes in myotubes. C2C12 cells were underwent skeletal muscle differentiation for seven days. RNA was extracted and analyzed for the indicated muscle-specific mRNAs by qPCR. Expression levels are shown relative to Gapdh. Due to the very large differences in expression, the data is plotted on a log scale. N=4-6. Genes were ascribed to skeletal muscle and/or heart muscle via the Human Protein Atlas.

Supplementary Table 1. Sequences for Figure 5C. The Sp3 binding peaks were identified as described. Five base-pair sequences surrounding the Sp3 binding peak are shown.

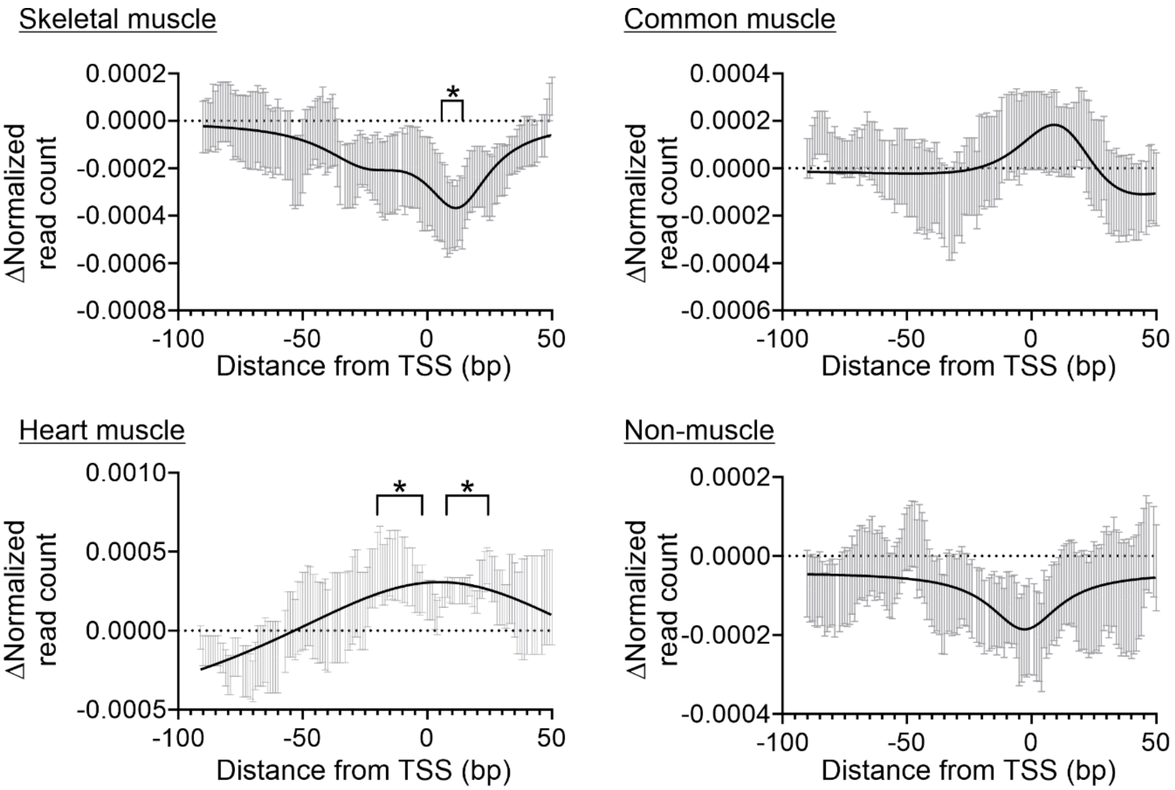

Supplementary Figure 1

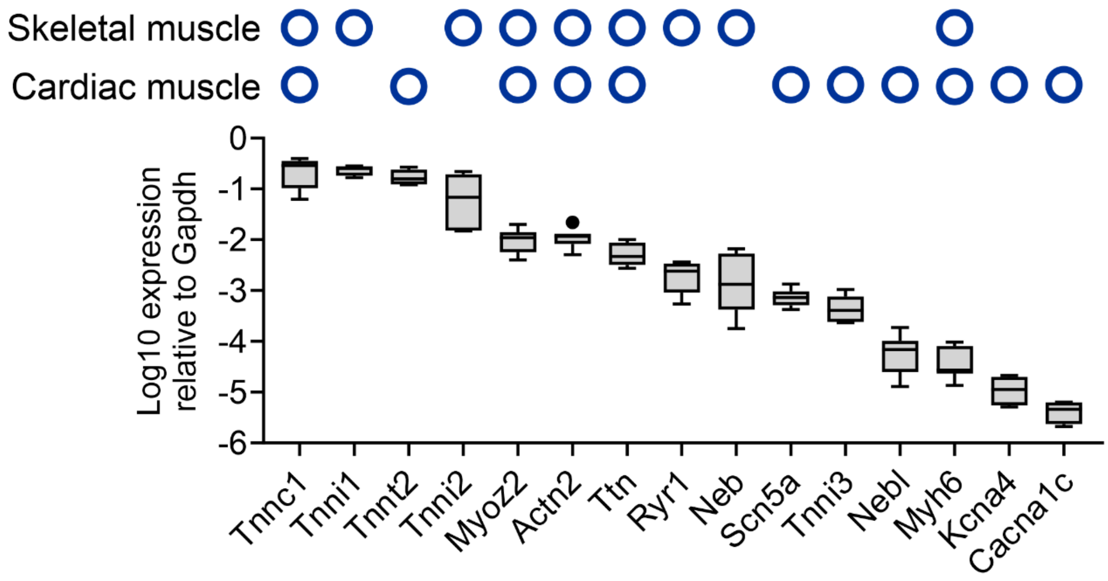

Supplementary Figure 2

Supplementary Table 1.

Muscle	Gene	Control (C) or Myotube (M)	Position relative to TSS	Sequence
Yes	Mef2C	M	-2685	C T G C C T C A C T C
Yes	Myh6	M	-2882	G T C G A A C T C G T
Yes	Tnni3	M	-2603	C C C G C T C C C A T
Yes	Cacna1c	C	-2957	G T C C C G T T T T G
Yes	Ryr2	M	-1781	A G T A T T T T C G A
Yes	Myl2	M	-899	T T G G G A G A C A G
Yes	Sln	M	-950	T T G T C T G A T T T
Yes	Kcnj2	M	+836	A C T C T C T A A T T
Yes	Kcna4	M	-2925	T T A T A A A A A A A
Yes	Neb	M	-1532	C T C G T C T A C T A
Yes	Ryr1	C	-494	C G T A G G T T C G A
Yes	Ryr1	M	-1511	C G A A A A T A A A A
Yes	Scn4a	C	+143	T A A A T T C T C G G
Yes	Scn4a	M	-3000	T C C C T T C A C C T
Yes	Cacna1s	C	+416	A A T C T C C A T C T
Yes	Cacna1s	M	-655	T T G G A G G A C C A
Yes	Tnni1	M	-1695	T T T G G T T T G T T
Yes	Tnni2	M	-16	T T T A T A C C C C T
Yes	Myod1	M	-2042	T G G C T C T C A G T
Yes	Myogenin	M	-2729	T C C C C A A G A C C
Yes	Myogenin	C	-2028	A G A C A G C A C T T
Yes	Myh2	M	-1999	C A T A C A A C A T C
Yes	Myh4	M	-2406	C A T G G C T G G A C
Yes	Myh4	C	-557	T C C T G T G G A A A
Yes	Myh7	M	-2636	T C T T A A G A G A A
No	Atl1	C	+850	C T T T T A C T C C A
No	Col1a1	C	-268	A A G T A C A A G G G
No	S100a4	C	-2593	G T C A T A G A T G A
No	Rbfox3	C	-737	T C T C T C T C T C T
No	Mapt	M	-1388	C T G G C T T G G A T
No	Mapt	C	-909	G C A C C T T A A A A
No	L1cam	C	-2089	G G G T G A A G G G G
No	Ncam1	C	-962	C C T G A C A G T G A
No	Vwf	M	-2666	T T A C T G G T G G C
No	Cdh1	C	-2880	T C T C C C A C C A C
No	Eng	M	-2352	C C T T G T T G C A T
No	Flt4	M	-1265	A G C C A G A C A G G
